# Application of an Imaging-Based Sum Score for Cerebral Amyloid Angiopathy to the General Population: Risk of Major Neurological Diseases and Mortality

**DOI:** 10.3389/fneur.2019.01276

**Published:** 2019-12-06

**Authors:** Pinar Yilmaz, Mohammad Arfan Ikram, Mohammad Kamran Ikram, Wiro J. Niessen, Anand Viswanathan, Andreas Charidimou, Meike W. Vernooij

**Affiliations:** ^1^Department of Epidemiology, Erasmus Medical Center, Rotterdam, Netherlands; ^2^Department of Radiology and Nuclear Medicine, Erasmus Medical Center, Rotterdam, Netherlands; ^3^Department of Neurology, Erasmus Medical Center, Rotterdam, Netherlands; ^4^Department of Medical Informatics, Erasmus Medical Center, Rotterdam, Netherlands; ^5^Department of Neurology, Massachusetts General Hospital Stroke Research Center, Harvard Medical School, Boston, MA, United States

**Keywords:** cerebral small vessel disease, cerebral amyloid angiopathy, sum score, MRI, cognition, stroke, dementia, mortality

## Abstract

**Objective:** To assess the relation between a sum score of imaging markers indicative of cerebral amyloid angiopathy (CAA) and cognitive impairment, stroke, dementia, and mortality in a general population.

**Methods:** One thousand six hundred twenty-two stroke-free and dementia-free participants of the population-based Rotterdam Study (mean age 73.1 years, 54.3% women) underwent brain MRI (1.5 tesla) in 2005–2011 and were followed for stroke, dementia and death until 2016–2017. Four MRI markers (strictly lobar cerebral microbleeds, cortical superficial siderosis, centrum semiovale perivascular spaces, and white matter hyperintensities) were combined to construct the CAA sum score, ranging from 0 to 4. Neuropsychological testing measured during the research visit closest to scan date were used to assess general cognitive function and cognitive domains. The associations of the CAA sum score with cognition cross-sectionally and with stroke, dementia, and mortality longitudinally were determined using linear regression and Cox proportional hazard modeling adjusted for age, sex, hypertension, cholesterol, lipid lowering medication, atrial fibrillation, antithrombotic medication and *APOE*-ε2/ε4 carriership. Additionally, we accounted for competing risks of death due to other causes for stroke and dementia, and calculated absolute risk estimates.

**Results:** During a mean follow-up of 7.2 years, 62 participants suffered a stroke, 77 developed dementia and 298 died. Participants with a CAA score of 1 showed a lower Mini-Mental-State-Exam (fully-adjusted mean difference −0.21, 95% CI (−0.42–0.00) compared to a score of 0. In general, for increased CAA scores we saw a lower g-factor. The age and sex-adjusted hazard ratios (HRs) per point increase of the CAA score were 1.41 for stroke (95% CI, 0.99–2.00), 1.19 for dementia (95% CI, 0.86–1.65), and 1.26 for mortality (95% CI, 1.07–1.48). The results for dementia and stroke risk did not differ after correcting for the competing risk of death. For all outcomes, higher CAA scores showed higher absolute risk estimates over 10 years.

**Conclusions:** Our results suggest that in this community-dwelling population, a higher CAA score is related to cognitive impairment and a higher risk of stroke, dementia, and death. The composite CAA score can be used to practically quantify the severity of vascular brain injury.

## Introduction

Cerebral amyloid angiopathy (CAA) is a frequent form of sporadic cerebral small vessel disease caused by accumulation of amyloid-ß in leptomeningeal and cortical vessels and capillaries (1, 2). The pathogenesis of CAA is complex and its consequences can result in cognitive impairment, dementia and stroke with a high recurrence rate of intracerebral hemorrhages (3, 4).

Brain imaging markers that reflect parenchymal damage caused by small vessel brain injury have shown to be useful in a clinical setting to diagnose CAA (2, 5) These markers visible on magnetic resonance imaging (MRI) include lobar cerebral microbleeds (CMB), cortical superficial siderosis (cSS), centrum semiovale perivascular spaces (CSO-PVS) and white matter hyperintensities of presumed vascular origin (henceforth WMH) (6, 7) Several markers individually are thought to reflect different types of small vessel disease. For CAA, cSS has shown to be highly indicative as a marker in not only patient cohorts, but also in population-based studies (8–10) Previous studies have used these individual CAA markers to determine their relation to neurological outcomes (stroke and dementia) and mortality in both healthy and diseased populations (9, 11–13).

Given that shared risk factors and pathophysiological pathways of CAA markers are known to overlap, and that often CAA patients have more than one of the brain imaging markers present, a recent study developed a composite CAA score by combining all four markers (CMB, cSS, CSO-PVS and WMH) (14). The authors concluded that this composite score may better reflect the overall CAA-related small vessel disease burden in the brain. Such composite scores reflecting CAA disease burden could be used in clinical practice or research settings (2, 14).

In a recent study, an association between the proposed CAA sum score and the prediction of dementia conversion in patients with probable CAA in absence of intracranial hemorrhage has been reported (15). Other studies in patients with CAA have shown that higher CAA scores are correlated with reduced global brain connectivity (16), and that patients who first present with transient focal neurological episodes have higher CAA scores than those who first present with cognitive complaints (17). In a patient population with ischemic cardioembolic stroke or transient ischemic attack (TIA) and non-valvular atrial fibrillation, patients who did not show improvement in their cognitive assessment in 12 months also had an increased CAA score (18).

Increasing evidence for the presence of subclinical CAA in the general population has been supported by high prevalence of lobar microbleeds identified in individuals over the age of 60 years, and the link with determinants such as APOE genotype similar to those found in CAA patients (4, 19–22). Though evidence is at present circumstantial, it is a logical next step to study whether presence of CAA markers in the general population leads to an increased risk of neurological events as well. This was recently shown for presence of microbleeds, with increased risk of cognitive decline, stroke, dementia and mortality (23–26). Despite the clear clinical potential of the developed CAA sum score, its correlations with major neurological outcomes and death have not yet been assessed in a general population. We therefore used data from the population-based Rotterdam Study to investigate the CAA score based on the four imaging markers in relation to cognitive status and risk of stroke, dementia, and mortality.

## Materials and Methods

### Study Participants

This study was conducted in the Rotterdam Study, a prospective population-based cohort study in which participants aged ≥45 years and living in the Ommoord district are examined and followed for various diseases (27). Imaging of the brain was incorporated in the study protocol from August 2005 onwards (28). For the present study, eligible participants of the fifth visit of the first wave (Rotterdam Study I-5), and second visit of the second wave (Rotterdam Study II-2) were included. 2015 out of 2,376 eligible participants (84.8%) underwent MRI in the period between 2005 and 2012. Participants were excluded if they had insufficient quality scans or missing sequences for ratings (*n* = 108), scans with missing PVS and CMB ratings (*n* = 94), and scans with MRI-defined large cortical infarcts (*n* = 53). In addition, participants without informed consent to access medical records and hospital discharge letters (*n* = 27) and if diagnosed with stroke or dementia or had incomplete follow-up for stroke and dementia diagnoses at time of MRI scan were excluded (*n* = 111). This resulted in 1,622 participants free from stroke and dementia with brain imaging data available for our analyses (Supplementary Figure 1).

The institutional review board (Medical Ethics Committee) approved the Rotterdam Study according to the Population Study Act, executed by the Ministry of Health, Welfare and Sports of the Netherlands. All participants gave written informed consent.

### MRI Scan Protocol and Assessment of CAA Imaging Markers

MRI of the brain was performed on a 1.5 tesla MRI scanner (GE-Healthcare). We acquired four high-resolution axial sequences without administering contrast material: T1-weighted sequence, proton density-weighted sequence, fluid-attenuated inversion recovery sequence and T2^*^-weighted gradient-recalled-echo sequence. Detailed information of the imaging protocol has been described elsewhere (28).

Trained research physicians rated the presence, number and location of CMB and PVS, and the presence of cSS and cortical infarcts on MRI. CMB were defined as focal areas <10 mm of very low signal intensity and rated on T2^*^-weighted imaging. We categorized CMB distribution based on their location in the brain into strictly lobar, lobar, and deep microbleeds. Strictly lobar microbleeds restricted to cortical gray matter and subcortical white matter, whereas lobar microbleeds could present with or without deep microbleeds. Cortical superficial siderosis was defined as linear hypointensities with gyriform patterns over the cerebral cortex on T2^*^-weighted images (8) PVS were defined as linear, ovoid or round-shaped hyperintensities of ≥1 and <3 mm and counted in the centrum semiovale, basal ganglia, hippocampi and midbrain on proton density-weighted images (29) PVS were counted on a single, predefined slice in the centrum semiovale (the slice 1 cm above the uppermost part of the lateral ventricles) and basal ganglia (the slice with the anterior commissure). For the hippocampi and midbrain, all PVS were counted in the anatomical areas. For this study we only used CSO-PVS and categorized the PVS in a validated visual rating scale of 0 to 4, defined as 0 = no PVS; 1 = <10 PVS; 2 = 11–20 PVS; 3 = 21–40 PVS and 4 = >40 PVS (30).

Quantitative measurements of WMH were obtained using a validated automated segmentation method (31). Intracranial volume (ICV) was defined as the summation of gray matter, white matter, and cerebrospinal fluid. Cortical infarcts were defined as focal lesions with tissue loss showing involvement of cortical gray matter.

### CAA Score

**Figure 1:**
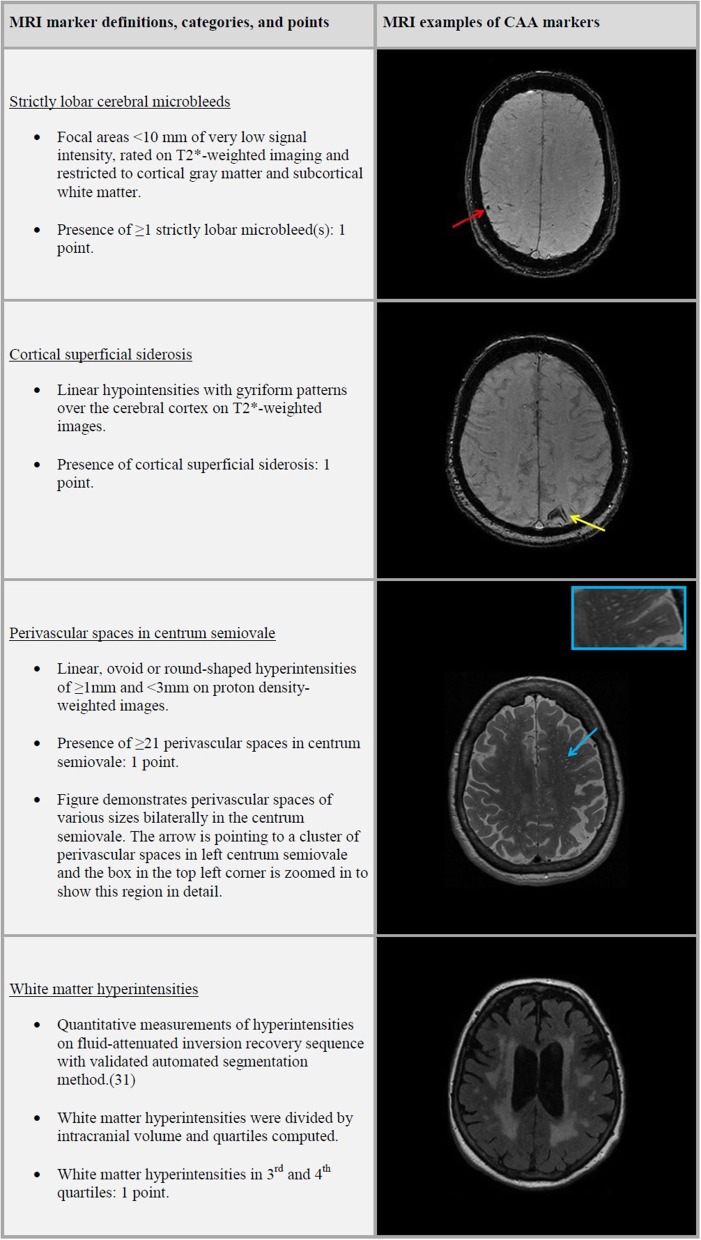
Magnetic Resonance Imaging (MRI) features of the cerebral amyloid angiopathy (CAA) score.

A simplified version of the CAA sum score (henceforth referred to as the CAA score) proposed by Charidimou et al. consisted of scoring the presence of strictly lobar CMB, cSS, CSO-PVS, and WMH, with a total score that ranged from 0 to 4 (Figure 1) (14). One point was given to the CAA score for presence of strictly lobar microbleeds and another point was given if any cSS was present. PVS categories of ≥21 CSO-PVS were awarded with one point to the score. We computed WMH quartiles after dividing total WMH volume by ICV. WMH burden in third or fourth quartiles were awarded with a point to the score.

### Modified Boston Criteria Score

In clinical practice, the modified Boston criteria is a tool to aid diagnose of CAA in patients. Therefore, we also explored the application of the modified Boston criteria in our population, and its relation with clinical outcomes. To operationalize this, the modified Boston criteria score was computed as an ordinal score ranging from 0 to 2. One point was given to this score if a single lobar microbleed was present or if cSS was present (representing possible CAA in the modified Boston criteria) (5). Two points were given to the modified Boston score if multiple lobar or cerebellar microbleeds were present or if a single lobar microbleed and cSS were present (i.e., probable CAA in the modified Boston criteria).

### Assessment of Cognitive Functioning

The cognitive assessment at one time-point, during the research visit closest to MRI date, included Mini-Mental State Examination (MMSE), letter-digit-substitution task (LDST), word fluency test (WFT), Stroop test, 15-word verbal learning test (15-WLT) and Perdue Pegboard test. For global cognition, we computed a standardized composite score (g-factor) with principal component analysis on the adjusted Stroop interference subtask, LDST, WFT, delayed recall of the 15-WLT, and Perdue Pegboard). The g-factor explained 43.1% of the total variance in cognitive test scores in our population. We combined different tests to construct compound scores for executive function (average Z-score of Stroop interference subtask, LDST, and WFT), information processing speed (average Z-score of Stroop reading and color-naming subtask, and LDST), memory (average Z-score of immediate and delayed recall of the 15-WLT), and motor speed (average Z-score of Perdue Pegboard test). New Z-scores were calculated for each compound score.

The median time between cognitive assessment and the MRI scan was 0.4 years (interquartile range: 0.1–1.0 years).

### Assessment of Stroke

History of stroke was assessed at study entry using home interviews and reviewing medical records. Stroke was defined as a syndrome of rapidly advancing clinical signs of focal or global disturbance of cerebral function lasting ≥24 h or cause death with no apparent cause other than of vascular origin, in accordance with World Health Organization criteria. Continuous monitoring for occurrence of stroke was realized through automated linkage of general practitioners' (GPs) files with the study database (32). Regular checking of medical files by contacting their treating physicians were done for participants who moved out of the district or into nursing homes. Research physicians reviewed all potential stroke cases using hospital discharge letters, information from GPs and from nursing home physicians. An experienced vascular neurologist verified the stroke diagnoses, these were classified as ischemic or hemorrhagic based on neuroimaging reports or hospital discharge letters and unspecified if these were absent.

Follow-up started on the date that participants received brain imaging. Participants were followed until date of stroke occurrence, date of death, date of last contact in case of loss to follow-up, or January 1st 2016, whichever came first. Follow-up was complete for 11564.8 (96.9%) of potential person-years.

### Assessment of Dementia

Participants were screened for dementia at baseline and during visits to the study center for incident and prevalent dementia. They underwent the MMSE and the Geriatric Mental Schedule (GMS). Subjects with MMSE <26 or GMS score>0 underwent further investigation and informant interview including the Cambridge Examination for Mental Disorders of the Elderly. Standard criteria were used for dementia (Diagnostic and Statistical Manual of Mental Disorders, version III, Revised), Alzheimer's disease (National Institute of Neurological and Communicative Disorders and Stroke-Alzheimer's Disease and Related Disorders Association), and vascular dementia (National Institute of Neurological Disorders and Stroke and Association Internationale pour la Recherché et l'Enseignement en Neurosciences). Continuous monitoring for dementia was accomplished through electronic linkage of the center with medical records from GPs and the regional institute for outpatient mental health care. Cognitive testing and clinical neuroimaging were used, if available, to diagnose dementia subtypes. Final diagnosis was made according to international criteria and established by a consensus panel led by a consultant neurologist (33).

Incident dementia follow-up started on the date that participants came for brain MRI. Follow-up of dementia was until January 1st 2016, participants were censored at date of dementia diagnosis, death, loss to follow-up, whichever came first. Until January 1st 2016, follow-up was complete for 11273.6 (94.9%) of potential person-years.

### Assessment of Mortality

Information on vital status of participants was collected from municipal health authorities in Rotterdam and updated for all-cause mortality on a monthly basis in the Rotterdam Study. Continuous reporting for incident events was achieved through automatic linkage of GPs files and verified by checking medical records to gather information on cause of death.

Participants were followed up from date of MRI scan until date of death, loss to follow-up or June 16th 2017, whichever came first. Follow-up was complete for 12110.2 (100%) of potential person-years.

### Assessments of Covariates

Participants were interviewed during center visits preceding brain MRI, and underwent laboratory and physical examinations for information on demographic, genetic and cardiovascular risk factors.

History of TIA and coronary heart disease, atrial fibrillation, diabetes mellitus, hypertension, serum total cholesterol, blood pressure and antithrombotic and lipid lowering medication, smoking, *APOE-*ε2/ε4 carriership, and education were used as covariates in this study. Definitions of included variables are presented in the online-only Supplementary Material.

### Statistical Analysis

Differences in baseline variables between CAA scores were assessed using chi-square test, Fisher's exact test or ANOVA. 5-fold multiple imputations were used for missing covariates [ranging from 0.001% (hypertension) to 10.6% (atrial fibrillation)] based on determinants, outcome status, and follow-up time. Distribution of covariates in imputed and non-imputed datasets showed no differences.

CAA scores of 3 and 4 occurred infrequently and were therefore combined. We investigated the CAA score and modified Boston criteria score in an ordinal manner and continuously in our analyses. Superficial siderosis and WMH were the strongest determinants amongst the individual CAA markers. We evaluated the dependency of the score on cSS and WMH by separately excluding one of the markers from the score, resulting in a maximum score of 3 in repeated analyses.

Multiple linear regression models were applied to investigate the cross-sectional association between the combined CAA score and cognitive functioning. Cox proportional-hazards models were used to investigate the association between the combined CAA score, the modified Boston criteria score and individual CAA markers with the risk of stroke, dementia, or mortality. All models were corrected for age and sex (model 1). Additionally, we corrected for hypertension, cholesterol, lipid lowering and antithrombotic medication, history of atrial fibrillation, and *APOE*-ε2/ε4 carriership based on literature (model 2) (1, 3). The proportional-hazards assumption was tested using Schoenfeld residuals and no violations were identified. Further, Fine-Gray modeling was used to perform competing risk analyses by modeling subdistribution hazards to assess mortality as a competing risk for stroke and dementia (34). Goodness-of-fit tests revealed no linear, quadratic or log time-varying effects of categorical and continuous CAA score (*p* > 0.1 for all analyses) for the Fine-Gray models. After adjusting for model 2, absolute risks of stroke and dementia were estimated up to 10 years with Fine-Gray modeling and for mortality with Cox modeling according to the CAA score. We used bootstrap resampling (*n* = 5,000) to estimate confidence intervals of the absolute risk estimates. Models did not converge for 1-year absolute risk estimates of stroke and dementia and 2-year risk estimates of stroke, due to low number of incident events and were excluded.

We studied the relation of the CAA score with subgroups of the neurological outcomes and mortality, namely ischemic and hemorrhagic stroke, Alzheimer's disease, and cardiovascular mortality. Finally, we explored non-linear effects of age in our models by adding age-squared or natural cubic splines with 3 degrees of freedom.

All analyses were performed using statistical software packages SPSS (version 24.0) and R using cmprsk (35), riskRegression (36), and nricens (37) packages (version 3.5.2, R Foundation for Statistical Computing, R Core Team (38), Vienna, Austria). The significance threshold was set at *P* < 0.05. The Strengthening the Reporting of Observational Studies in Epidemiology statement was used as guideline (39).

## Results

### Baseline Characteristics

Characteristics of the study population are shown in Table 1. Of the total of 1,622 participants, 54.3% were women and the average age at baseline was 73.1 years (SD 7.6). The majority of the participants had a CAA score of 1 (*n* = 753) and only one participant had a score of 4. More men than women had scores of 1 and 2, and women more often had CAA scores of 0 and 3 (*p* = 0.002). The MMSE score ranged between 12.0 and 30.0 (median 28.0). Mean follow-up time was 7.2 years for stroke, dementia and death. In our study, 62 participants suffered from a stroke, 77 developed dementia and 298 died.

**Table 1:** Baseline characteristics of the study population.

	**All participants, *N* = 1622**
Age, years	73.1 (7.6)
Female sex, *n*	880 (54.3)
History of transient ischemic attack, *n*	110 (6.8)
History of coronary heart disease, *n*	146 (9.0)
History of atrial fibrillation^ǂ^, *n*	86 (5.9)
History of diabetes mellitus^ǂ^, *n*	185 (11.6)
Hypertension^ǂ^, *n*	1,276 (78.8)
Systolic blood pressure^ǂ^, mmHg	148.5 (20.7)
Diastolic blood pressure^ǂ^, mmHg	82.5 (10.8)
Body mass index, kg/m^2^	27.4 (3.8)
Total cholesterol^ǂ^, mmol/L	5.5 (1.0)
Blood pressure lowering medication^ǂ^, *n*	756 (46.7)
Antithrombotic medication^ǂ^, *n*	454 (28.1)
Serum lipid lowering medication^ǂ^, *n*	441 (27.3)
Smoking status^ǂ^, *n*	
Never	513 (31.9)
Current	221 (13.7)
Former	875 (54.4)
*APOE-*ε2/ε4 carrier^ǂ^, *n*	643 (40.5)
Education^ǂ^, years	12.3 (3.7)
Imaging markers	
Strictly lobar cerebral microbleeds, *n*	284 (17.5)
Cortical superficial siderosis, *n*	10 (0.6)
Centrum semiovale perivascular spaces, *n*	1,490 (93.0)
0	113 (7.0)
≤10	1,120 (69.1)
≥11–20	316 (19.5)
≥21–40	73 (4.5)
WMH, mL^*^	4.4 [2.4–9.1]
First quartile	0.4–2.4
Second quartile	2.5–4.4
Third quartile	4.5–9.1
Fourth quartile	9.2–135.1

*Non-imputed values are means (standard deviation) or numbers (valid percentages)*.

* *Intracranial volume-corrected white matter hyperintensities (WMH) shown in median and interquartile range with separate quartile ranges*.

ǂ *Data was missing for the following variables: history of atrial fibrillation (10.6%), history of diabetes mellitus (1.8%), hypertension (0.1%), systolic blood pressure (0.1%), diastolic blood pressure (0.5%), total cholesterol (0.4%), smoking status (0.8%), blood pressure lowering medication (0.2%), antithrombotic medication (0.6%), serum lipid lowering medication (0.5%), APOE-ε2/ε4 carriership (2.2%), and education (1.9%)*.

### Association of CAA Score With Cognitive Functioning

**Figure 2:**
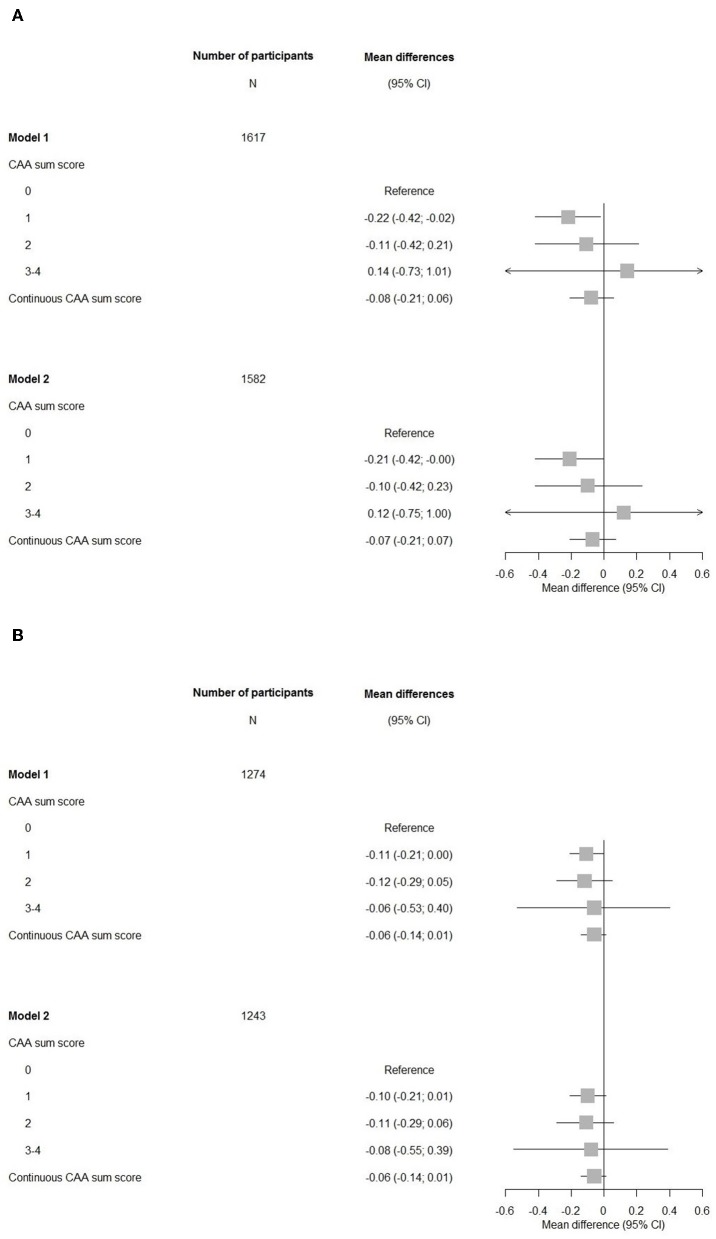
Forest plots of associations between the cerebral amyloid angiopathy score and cognitive measures. CI, confidence interval; CAA, cerebral amyloid angiopathy. Model 1: adjusted for age and sex. Model 2: adjusted for age, sex, hypertension, cholesterol, lipid lowering medication, history of atrial fibrillation, antithrombotic medication, and *APOE*-ε2/ε4 carriership. **(A)** Mini-Mental State Examination. **(B)** G-factor.

Participants with a CAA score of 1 showed a significant lower MMSE after adjustments for cardiovascular factors and *APOE-*ε2/ε4 carriership compared to those with a score of 0 (mean difference −0.21, 95% CI (−0.42–0.00), Figure 2). An increasing CAA score related to a lower g-factor, yet none of the associations with g-factor were significant.

**Figure 3:**
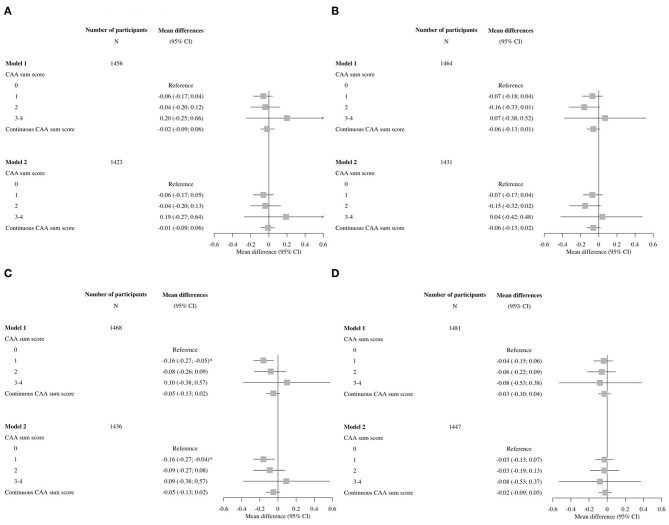
Forest plots of associations between the cerebral amyloid angiopathy score and specific cognitive domains. CI, confidence interval; CAA, cerebral amyloid angiopathy. Model 1: adjusted for age and sex. Model 2: adjusted for age, sex, hypertension, cholesterol, lipid lowering medication, history of atrial fibrillation, antithrombotic medication and *APOE*-ε2/ε4 carriership. **(A)** Executive function, **(B)** information processing speed, **(C)** memory, and **(D)** motor speed.

Overall, the associations of cognitive domains were also not significant, only for the memory domain, having a CAA score of 1 compared to a score of 0 showed a significant impairment persisting after further adjustments in model 2 (Figure 3).

### Association of CAA Score With Stroke, Dementia, and Mortality

Higher CAA scores were related to higher HRs for stroke, dementia and mortality (Table 2). After further adjustments in model 2, having a score of 1 remained significant for stroke. Risk for developing dementia was highest for participants with CAA scores of 3–4 and remained significant after correction in model 2 [HR 3.25, 95% CI (1.00–10.54)]. When comparing subjects with a CAA score of 2 compared to a score of 0, associations were seen with mortality after additional adjustments for cardiovascular risk factors and *APOE-*ε2/ε4 carriership.

**Table 2:** The association of the cerebral amyloid angiopathy score with stroke, dementia, and mortality.

	***N***	***n***	**Stroke** **HR (95%CI)**	***n***	**Dementia** **HR (95%CI)**	***n***	**Mortality** **HR (95%CI)**
**Model 1**							
**CAA score**							
0	666	14	1.00 (reference)	20	1.00 (reference)	76	1.00 (reference)
1	753	38	2.22 (1.17–4.21)^*^	45	1.45 (0.83–2.51)	162	1.34 (1.00–1.78)^*^
2	185	9	2.08 (0.86–5.02)	8	0.86 (0.37–2.02)	53	1.66 (1.15–2.40)^*^
3–4	18	1	2.04 (0.26–15.95)	4	4.61 (1.51–14.08)^*^	7	1.70 (0.77–3.74)
Continuous CAA score							
	1,622	62	1.41 (0.99–2.00)	77	1.19 (0.86–1.65)	298	1.26 (1.07–1.48)^*^
**Model 2**^ǂ^							
**CAA score**							
0	651	13	1.00 (reference)	18	1.00 (reference)	75	1.00 (reference)
1	738	38	2.18 (1.13–4.22)^*^	44	1.52 (0.85–2.72)	160	1.28 (0.96–1.71)
2	180	9	2.01 (0.82–4.92)	8	0.90 (0.38–2.15)	51	1.49 (1.02–2.17)^*^
3–4	18	1	1.48 (0.18–11.89)	4	3.25 (1.00–10.54)^*^	7	1.34 (0.60–3.00)
Continuous CAA score							
	1,587	61	1.33 (0.93–1.89)	74	1.16 (0.84–1.60)	293	1.18 (1.00–1.39)^*^

*N, number of participants; n, number of events; HR, hazard ratio; CI, confidence interval; CAA, cerebral amyloid angiopathy*.

*Model 1: adjusted for age and sex*.

*Model 2: adjusted for age, sex, hypertension, cholesterol, lipid lowering medication, history of atrial fibrillation, antithrombotic medication, and APOE-ε2/ε4 carriership*.

ǂ *Data missing for APOE-ε2/ε4 carriership n = 35*.

* *P < 0.05*.

Excluding cSS from the combined CAA score attenuated associations of the scores for all outcomes and only remained significant for score 1 and MMSE and score 1 and stroke after further adjustments (model 2, Table 3 and Supplementary Table 2). After excluding WMH from the CAA score, all associations again attenuated and none remained significant (Supplementary Tables 3, 4).

**Table 3:** The association of cerebral amyloid angiopathy score excluding cortical superficial siderosis with stroke, dementia and mortality.

	***N***	***n***	**Stroke** **HR (95%CI)**	***n***	**Dementia** **HR (95%CI)**	***n***	**Mortality** **HR (95%CI)**
**Model 1**							
**CAA score excluding cSS**							
0	667	15	1.00 (reference)	20	1.00 (reference)	77	1.00 (reference)
1	755	38	2.05 (1.09–3.82)^*^	45	1.44 (0.83–2.50)	162	1.32 (0.99–1.75)
2–3	200	9	1.73 (0.72–4.12)	12	1.18 (0.56–2.53)	59	1.62 (1.13–2.32)^*^
Continuous CAA score excluding cSS							
	1,622	62	1.37 (0.94–2.02)	77	1.12 (0.79–1.59)	298	1.27 (1.07–1.52)^*^
**Model 2**^ǂ^							
**CAA score excluding cSS**							
0	652	14	1.00 (reference)	18	1.00 (reference)	76	1.00 (reference)
1	740	38	2.00 (1.05–3.80)^*^	44	1.50 (0.84–2.68)	160	1.26 (0.95–1.68)
2–3	195	9	1.60 (0.66–3.88)	12	1.18 (0.54–2.57)	57	1.43 (0.99–2.06)
Continuous CAA score excluding cSS							
	1,587	61	1.31 (0.89–1.94)	74	1.11 (0.78–1.59)	293	1.20 (1.00–1.44)

*N, number of participants; n, number of events; HR, hazard ratio; CI, confidence interval; CAA, cerebral amyloid angiopathy; cSS, cortical superficial siderosis*.

*Model 1: adjusted for age and sex*.

*Model 2: adjusted for age, sex, hypertension, cholesterol, lipid lowering medication, history of atrial fibrillation, antithrombotic medication and APOE-ε2/ε4 carriership*.

ǂ *Data missing for APOE-ε2/ε4 carriership n = 35*.

* *P < 0.05*.

### Association of the Modified Boston Criteria Score With Stroke, Dementia, and Mortality

The modified Boston criteria score showed higher risk for developing dementia with a score of 2 (probable CAA) and for death with a score of 1 (possible CAA) after adjusting for cardiovascular risk factors and *APOE-*ε2/ε4 carriership (Supplementary Table 5). The HRs of the Boston criteria score were lower compared to the CAA score, particularly for stroke.

### Association of Individual CAA MRI Markers With Stroke, Dementia, and Mortality

CSO-PVS, cSS, and WMH were related to all outcomes (Table 4). Strictly lobar microbleeds were only related with mortality. Ten participants had superficial siderosis and this marker showed the highest risk among all markers for all outcomes. Participants with cSS had a 2.2–5.5 times higher risk of developing an event in our population. These relations remained significant for stroke and mortality after correcting for cardiovascular risk factors and *APOE-*ε2/ε4 carriership. Other significant associations were found for WMH and stroke (model 2).

**Table 4:** The association of cerebral amyloid angiopathy MRI markers with stroke, dementia and mortality.

	***N***	***n***	**Stroke** **HR (95%CI)**	***n***	**Dementia** **HR (95%CI)**	***n***	**Mortality** **HR (95%CI)**
**Strictly lobar cerebral microbleeds**							
None^a^			1.00 (reference)		1.00 (reference)		1.00 (reference)
≥1, model 1	284	8	0.64 (0.30–1.34)	15	0.96 (0.54–1.70)	67	1.17 (0.89–1.54)
≥1, model 2^ǂ^	277	8	0.61 (0.29–1.30)	15	0.95 (0.54–1.69)	65	1.10 (0.83–1.46)
**Cortical superficial siderosis**							
None			1.00 (reference)		1.00 (reference)		1.00 (reference)
Present, model 1	10	3	7.11 (2.17–23.33)^*^	3	4.88 (1.51–15.78)^*^	8	2.66 (1.31–5.43)^*^
Present, model 2^ǂ^	10	3	5.49 (1.56–19.35)^*^	3	3.07 (0.90–10.42)	8	2.16 (1.03–4.54)^*^
**Centrum semiovale perivascular spaces**							
≤20			1.00 (reference)		1.00 (reference)		1.00 (reference)
≥21, model 1	73	4	1.34 (0.49–3.70)	5	1.19 (0.48–2.95)	20	1.18 (0.74–1.87)
≥21, model 2^ǂ^	72	4	1.27 (0.46–3.53)	5	1.14 (0.46–2.86)	20	1.18 (0.74–1.88)
**White matter hyperintensities**							
1st and 2nd quartiles			1.00 (reference)		1.00 (reference)		1.00 (reference)
3rd and 4th quartiles, model 1	811	44	2.29 (1.27–4.13)^*^	51	1.35 (0.81–2.25)	195	1.34 (1.04–1.72)^*^
3rd and 4th quartiles, model 2^ǂ^	794	44	2.19 (1.19–4.02)^*^	50	1.35 (0.79–2.30)	191	1.24 (0.96–1.60)

*MRI, magnetic resonance imaging; N, number of participants; n, number of events; HR, hazard ratio; CI, confidence interval*.

*Model 1: adjusted for age and sex*.

*Model 2: adjusted for age, sex, hypertension, cholesterol, lipid lowering medication, history of atrial fibrillation, antithrombotic medication and APOE-ε2/ε4 carriership*.

a *None strictly microbleeds include no microbleeds and microbleeds at other locations like deep and infratentorial microbleeds*.

ǂ *Data missing for APOE-ε2/ε4 carriership n = 35*.

* *P < 0.05*.

### Association of CAA Score With Stroke and Dementia Adjusted for the Competing Risk of Mortality and Absolute Risk Estimations for All Outcomes

The associations between the CAA score and stroke and dementia slightly attenuated after correcting for the competing risk of death (Table 5).

**Table 5:** The association of cerebral amyloid angiopathy score with stroke and dementia after adjusting for the competing risk of mortality.

	***N***	***n***	**Stroke** **sHR (95%CI)**	***n***	**Dementia** **sHR (95%CI)**
**Model 1**					
**CAA score**					
0	666	14	1.00 (reference)	20	1.00 (reference)
1	753	38	2.19 (1.14–4.22)^*^	45	1.39 (0.78–2.47)
2	185	9	2.00 (0.81–4.95)	8	1.24 (0.34–1.95)
3–4	18	1	2.02 (0.27–15.37)	4	4.56 (1.30–16.07)
Continuous CAA score					
	1,622	62	1.39 (1.01–1.90)^*^	77	1.17 (0.81–1.67)
**Model 2**^ǂ^					
**CAA score**					
0	651	13	1.00 (reference)	18	1.00 (reference)
1	738	38	2.17 (1.09–4.30)^*^	44	1.46 (0.79–2.69)
2	180	9	1.93 (0.77–4.84)	8	0.85 (0.34–2.12)
3–4	18	1	1.59 (0.21–12.09)	4	3.40 (0.87–13.29)
Continuous CAA score					
	1,587	61	1.32 (0.96–1.82)	74	1.14 (0.80–1.64)

*N, number of participants; n, number of events; sHR, subdistribution hazard ratio; CI, confidence interval; CAA, cerebral amyloid angiopathy*.

*Model 1: adjusted for age and sex*.

*Model 2: adjusted for age, sex, hypertension, cholesterol, lipid lowering medication, history of atrial fibrillation, antithrombotic medication and APOE-ε2/ε4 carriership*.

ǂ *Data missing for APOE-ε2/ε4 carriership n = 35*.

* *P < 0.05*.

**Figure 4:**
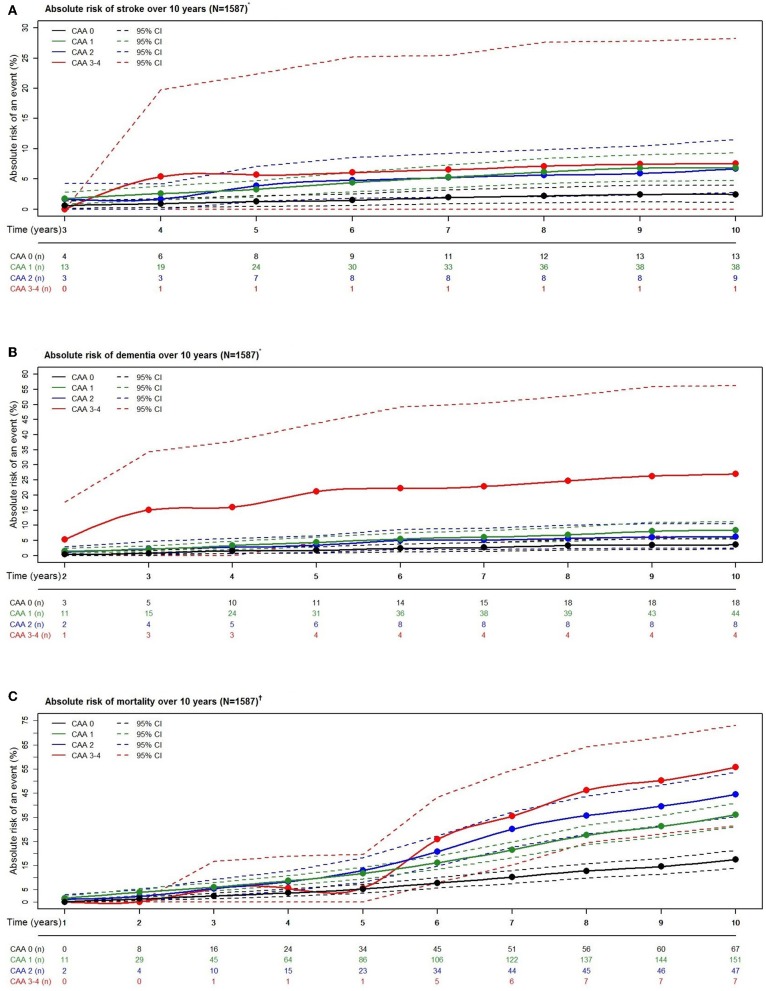
Absolute risk estimates of stroke **(A)**, dementia **(B)**, and mortality **(C)** according to cerebral amyloid angiopathy score category over a period of 10 years. N, number of participants; n, number of events. *Estimates calculated with competing risk modeling. ^†^Estimates calculated with Cox modeling. *^†^Adjusted for age, sex, hypertension, cholesterol, lipid lowering medication, history of atrial fibrillation, antithrombotic medication, and *APOE*-ε2/ε4 carriership.

For all outcomes over 10 years, higher CAA scores noted increased risk estimates (Figure 4).

Subgroup and sensitivity analyses are presented in the online Supplementary Material.

## Discussion

We found that a higher imaging-based CAA score relates to cognitive impairment and a higher risk of stroke, dementia, and mortality in our community-dwelling population. Our study is the first study to apply the proposed CAA sum score in a non-diseased population.

Previous studies have investigated the CAA score in patients with CAA (or probable CAA), ischemic cardioembolic stroke, or TIA with non-valvular atrial fibrillation (14–18). Patients with an ischemic cardioembolic stroke or TIA with atrial fibrillation were also more likely to have higher CAA scores and persistent cognitive impairment over a 12-month period (18). They found the CAA score to be associated to cognitive impairment, although the sample size of the study was small (*N* = 117). We found a relation with having a CAA score of 1 and a significant lower MMSE and a significant impairment of the memory domain with a trend of an increasing CAA score leading to a lower g-factor. Even though these observations were cross-sectional and overall not significant, they were in the direction of our hypothesis. Lower test scores for memory, executive function and processing speed have previously been described in patients with CAA without dementia (40). The pattern we observed for cognitive impairment in our population could have several explanations. Application of the CAA score to a general population could reflect non-CAA etiology compared to applying the score in a population with CAA patients and not lead to the expected pattern of cognitive impairment as seen in CAA pathology. Another explanation could be the long latency period of CAA for cognitive changes in a subclinical population.

In our study, of all the outcomes, dementia had the strongest association with the CAA score after adjusting for cardiovascular factors and *APOE*-ε2/ε4 carriership [HR 3.25, 95% CI (1.00–10.54)]. The study by Banerjee et al. (18) also examined the cerebral small vessel disease (CSVD) score in patients with atrial fibrillation-related ischemic stroke or TIA. The CSVD score is a more well-known sum score to capture the global burden of small vessel disease in the brain (41). Previously, we also investigated the CSVD score in the general population and found that stroke was correlated strongest with this score amongst the outcomes for stroke, dementia and mortality in our population [adjusted for the Framingham Stroke Risk Profile, HR 3.47 (1.33–9.06)] (42). Since it is hypothesized that composite scores reflect the overall disease burden better, this can indicate that different combinations of individual markers reflect different pathological mechanisms even when markers co-occur in the disease spectrum. CSVD is a spectrum referring to a group of pathological processes with different mechanisms affecting small arteries, veins and capillaries in the brain (43). Since these pathologies partially co-occur and lead to similar markers in different types of CSVD, there might be an overlap between CSVD types and with CSVD and CAA scores and we might not capture the pure disease type. Yet, depending on the setting, to distinguish the pure disease type would be more desirable for a clinical setting and to capture broad CSVD would be more useful in a population-based setting. Conversely, CSVD, and CAA scores are more likely to reach a ceiling effect within patient populations, losing the granularity of individual markers, whereas in a population-based setting they may better capture the cumulative effect and variability of subclinical disease. Another way these scores could assist, is to detangle individual and shared underlying mechanisms of CSVD biomarkers. Moreover, reliable identification of asymptomatic CSVD with specific markers could aid in selecting high risk individuals, monitoring CSVD progression and predicting conversion of individuals from asymptomatic to symptomatic CSVD (44).

Interestingly, we found no relation of strictly lobar microbleeds with stroke and dementia, whereas we previously found this in a larger population with microbleeds (23, 24). There are several possible explanations for this. Firstly, the definitions used for microbleeds differ, i.e., we separately categorized strictly lobar microbleeds in the current study whereas in previous studies “CAA-related” microbleeds were defined, which include lobar microbleeds with or without cerebellar microbleeds. Secondly, we observed a small number of events per outcome for the presence of strictly lobar microbleeds. The third explanation is that microbleeds might be more acute markers and its subclinical impact varies over time. Another study found that while presence of strictly lobar microbleeds reflects CAA in individuals without intracerebral hemorrhage, the diagnostic accuracy of lobar microbleeds in the general population was limited (45).

The modified Boston criteria are diagnostic criteria often used in clinical practice and applied to identify other CAA biomarkers in research (5). Application of these criteria in a non-selected population should not be compared to its use in a clinical setting, but we consider it of value to compare the performance of the CAA score to the modified Boston criteria in terms of risk prediction. In our population-based study, we found that the CAA score estimated the relative risks for major neurological outcomes and death better than the modified Boston criteria. Recently, a review that summarized the validation of the Boston criteria for probable CAA concluded that in a community-based cohort the sensitivity was very low relative to hospital-based cohorts (5, 45). This finding assumes that the current set of criteria is insufficient to adequately identify probable CAA in a subclinical setting. Presumably, CAA pathology has a latent period during which CAA-related damage advances prior to becoming severe enough to be diagnosed (5). Improving the Boston criteria by incorporating non-hemorrhagic imaging markers to better reflect CAA pathology on imaging is currently a key issue for the future directions in diagnosis of CAA (2, 5). Examples of such imaging markers are CSO-PVS, WMH and cortical microinfarcts, or more advanced imaging markers derived from diffusion tensor imaging (5, 7). The diagnostic accuracy of the CAA score in combination with pathological verification and the added value of new markers should be evaluated to establish its practical use in research and in clinics.

The strengths of our study are the longitudinal population-based design and extensive data collection. However, several limitations should also be considered. First, the CAA sum score proposed by Charimidou et al. (14) used visual ratings of WMH, whereas we used quantitative WMH volumes. Despite this, quantitative and qualitative WMH assessments have been shown to be acceptable in a population-based setting (46). Although, we used our volumetric WMH data to approximate the Fazekas scale, for clinical use a semi-quantitative rating with the Fazekas scale would be more practical, as described in the original CAA score (14, 47). Second, our CAA score was defined as a simple addition of dichotomized imaging markers with certain thresholds, which presume arbitrary cut-offs and may not reflect true biological processes. Specifically, cut-offs based on counts could depend on imaging techniques or scanner parameters, for example markers like CMB and PVS. Potentially, using other methods to combine imaging biomarkers, e.g., using machine learning techniques, could have led to more informative markers and increased statistical power. Yet, point-based scores such as the CAA sum score are likely to be more practical in clinical and trial settings. Third, the CAA score had an uneven distribution with fewer events in participants with CAA scores of 3–4 due to the small numbers of events in the long-term follow-up. Fourth, we investigated cognitive deterioration in a cross-sectional design with MMSE and g-factor which are crude global measures of cognition. Longitudinal research will provide more robust results and gain more insight in these findings. Also, healthier participants without subjective memory complaints are more likely to receive cognitive retesting during the study follow-up which may have led to selection bias and influenced our results. Nonetheless, in that case our results presumptively would be biased toward the null. Fifth, although we aimed to address potential confounders based on the literature, residual confounding and unmeasured confounders may have affected our results to some extent. Sixth, a definitive diagnosis of CAA relies on pathological examination, yet obtaining pathological confirmation was not feasible in our population-based setting. Lastly, the majority of our population is Caucasian and generalizability of our study results to other ethnicities is therefore limited.

## Conclusions

The results of this study in a community-dwelling population indicate that a higher CAA score is related to cognitive impairment and a higher risk of stroke, dementia, and mortality. Our findings suggest that the practical use of the CAA score to quantify the severity of vascular brain injury in an elderly population could further assist etiologic or predictive research purposes. Further evaluation of the score is needed to establish its application in clinical practice.

## Data Availability Statement

Data can be obtained upon request. Requests should be directed toward the management team of the Rotterdam Study (secretariat.epi@erasmusmc.nl), which has a protocol for approving data requests. Because of restrictions based on privacy regulations and informed consent of the participants, data cannot be made freely available in a public repository.

## Ethics Statement

The studies involving human participants were reviewed and approved by The institutional review board (Medical Ethics Committee) approved the Rotterdam Study according to the Population Study Act, executed by the Ministry of Health, Welfare and Sports of the Netherlands. The participants provided their written informed consent to participate in this study.

## Author Contributions

PY, AC, AV, and MV: conception and design of the research. PY, MKI, MAI, and MV: acquisition of the data. PY, MAI, AC, AV, and MV: analysis and interpretation of the data. PY: drafting the manuscript. PY, MAI, MKI, WN, AV, AC, and MV: critical revision of the manuscript. PY, MAI, MKI, WN, AV, AC, and MV: final approval of the version to be published.

### Conflict of Interest

The authors declare that the research was conducted in the absence of any commercial or financial relationships that could be construed as a potential conflict of interest.
