# Supplementary material for: Application of an Imaging-Based Sum Score for Cerebral Amyloid Angiopathy to the General Population: Risk of Major Neurological Diseases and Mortality
Source: Front Neurol. 2019 Dec 6;10:1276. doi: 10.3389/fneur.2019.01276 (PMC6908500; doi:10.3389/fneur.2019.01276)
Supplement: Supplementary file 1 [file Presentation_1.pdf]

SUPPLEMENTAL MATERIAL

Application of an imaging-based sum score for cerebral amyloid angiopathy to the general population: risk of major neurological diseases and mortality.

Pinar Yilmaz, MD MSc[1,2]; M. Arfan Ikram, MD PhD[1]; M. Kamran Ikram, MD PhD[1,3]; Wiro J. Niessen, PhD[2,4]; Anand Viswanathan, MD PhD[5]; Andreas Charidimou, MD PhD[5]; Meike W. Vernooij, MD PhD[1,2]*

[1]Department of Epidemiology, Erasmus Medical Center, Rotterdam, the Netherlands
[2]Department of Radiology and Nuclear Medicine, Erasmus Medical Center, Rotterdam, the Netherlands
[3]Department of Neurology, Erasmus Medical Center, Rotterdam, the Netherlands
[4]Department of Medical Informatics, Erasmus Medical Center, Rotterdam, the Netherlands
[5]Department of Neurology, Massachusetts General Hospital Stroke Research Center, Harvard Medical School, Boston, Massachusetts

*Correspondence to: Professor Meike W. Vernooij, Erasmus Medical Center, Departments of Radiology and Epidemiology P.O. Box 2040, 3000 CA, Rotterdam, the Netherlands. Phone +31-10-7030944, Fax +31-10-7035372, E-mail m.vernooij@erasmusmc.nl.

Supplemental Appendix

Supplemental Methods.	Page 3
Supplemental Results.	Page 4
Supplementary Table I. The association of cerebral amyloid angiopathy score with cognitive tests.	Page 5
Supplementary Table II. The association of cerebral amyloid angiopathy score excluding cortical superficial siderosis with general cognition.	Page 7
Supplementary Table III. The association of cerebral amyloid angiopathy score excluding white matter hyperintensities with general cognition.	Page 9
Supplementary Table IV. The association of cerebral amyloid angiopathy score excluding white matter hyperintensities with stroke, dementia and mortality.	Page 10
Supplementary Table V. The association of the cerebral amyloid angiopathy score according to the modified boston criteria with stroke, dementia and mortality.	Page 11
Supplementary Table VI. STROBE statement checklist.	Page 12
Supplementary Figure I. Flowchart of study population.	Page 14
Supplemental References.	Page 16

Supplemental Methods

Assessments of covariates

Participants were interviewed for history of TIA which were verified using medical records. Research physicians reviewed potential TIA cases that were collected from general practitioners and hospital records. TIA diagnoses were verified by an experienced vascular neurologist according to the WHO criteria.(1) Prevalent coronary heart disease was defined as having had a myocardial infarction or a surgical/percutaneous revascularization procedure. Coronary heart disease and atrial fibrillation were assessed using established standardized definitions.(2) Diabetes mellitus was defined as having a fasting glucose level of ≥7.0 mmol/L or the use of antidiabetic medication. Blood pressure measurements were averaged over two readings using a random-zero sphygmomanometer. Hypertension was defined as ≥140/90 mm Hg or the use of blood pressure–lowering medication. Serum total cholesterol was measured using an automated enzymatic procedure (Boehringer Mannheim System). Blood pressure, antithrombotic and lipid lowering medication use were assessed during home interviews. Smoking habits were classified as never, former and current smokers. *APOE*-ε2/ε4 carriership was determined using a polymerase chain reaction (PCR) and TaqMan assay on coded genomic deoxyribonucleic acid (DNA) samples.(3) Haplotype Reference Consortium (HRC) imputed genotype values for rs7412 and rs429358 were used to define the *APOE*-ε2/ε4 carriership if these values were missing with the previous methods. Education was derived from self-reported history and scaled in number of years according to the UNESCO classification.(4)

Supplemental results

Association of CAA score with stroke and dementia adjusted for the competing risk of mortality and absolute risk estimations for all outcomes

A twofold increased risk of stroke was seen in score 1 when compared to score 0 (Figure 4). For dementia, scores of 3-4 showed an increased risk of up to ten times when compared to scores 0, 1 and 2. After 5 years, scores of 1 and 2 showed significant higher risk of dying compared to a score of 0. Scores of 3-4 showed significant associations for mortality after 6.5 years compared to a score of 0.

Association of CAA score with cognitive tests

Individual cognitive tests revealed worse performance on the Stroop interference test and the delayed word learning test with a CAA score of 1, these associations persisted with further adjustments in model 2 (Supplementary Table 1).

Subgroup analyses

In our study population, 48 participants had ischemic strokes and 13 hemorrhagic strokes, further 63 participants developed Alzheimer's disease and one vascular dementia. A total of 31 out of 298 deaths were cardiovascular related deaths. CAA score of 1 was related to ischemic stroke and a score of 3-4 was related to Alzheimer's disease after adjusting for age and sex. However, after further adjustments the associations attenuated and were not significant (model 2). We found similar associations for the ordinal and continuous CAA score excluding cSS and the outcomes. Exclusion of WMH from the CAA score showed similar associations as described in Supplementary Table 2, except the hazards were higher for hemorrhagic stroke compared to ischemic stroke. After correction for cardiovascular risk factors and APOE-ε2/ε4 carriership, the continuous CAA score for ischemic and hemorrhagic stroke showed HR 0.71, 95% CI (0.36-1.41) and HR 2.11, 95% CI (0.85-5.23) , respectively. Also with the modified Boston criteria score, the risk for hemorrhagic stroke was higher than for ischemic stroke. Overall, the risk of cardiovascular death was slightly higher compared to the risk of mortality presented in Table 3 when excluding cSS or WMH from the CAA score. Data not shown for subtype analyses.

Sensitivity analyses

Adding age squared or cubic splines to assess the non-linear relation of age with the outcomes did not change the effect of age in our models (data not shown).

Supplemental Tables

Supplementary Table I. The association of cerebral amyloid angiopathy score with cognitive tests.

Mean differences (95% CI)	Letter-digit substitution test (N=1569)	Stroop interference test (N=1493)	Word fluency test (N=1545)	Word learning test delayed (N=1469)	Perdue pegboard test (N=1440)
Model 1					
CAA score					
0	Reference	Reference	Reference	Reference	Reference
1	-0.10 (-0.20; 0.00)	-0.15 (-0.26; -0.04)*	0.023 (-0.08; 0.14)	-0.13 (-0.25; -0.02)*	-0.06 (-0.16; 0.04)
2	-0.13 (-0.29; 0.03)	-0.15 (-0.32; 0.02)	0.12 (-0.05; 0.29)	-0.07 (-0.24; 0.11)	-0.06 (-0.22; 0.09)
3-4	0.23 (-0.21; 0.67)	0.12 (-0.33; 0.57)	-0.03 (-0.51; 0.44)	0.03 (-0.44; 0.51)	-0.20 (-0.63; 0.23)
Continuous CAA score	-0.05 (-0.12; 0.02)	-0.07 (-0.15; 0.00)	0.04 (-0.03; 0.12)	-0.05 (-0.12; 0.03)	-0.04 (-0.11; 0.02)
Model 2[‡]					
CAA score					
0	Reference	Reference	Reference	Reference	Reference
1	-0.10 (-0.20; 0.01)	-0.15 (-0.26; -0.03)*	0.03 (-0.08; 0.14)	-0.13 (-0.25; -0.02)*	-0.04 (-0.14; 0.06)
2	-0.12 (-0.28; 0.05)	-0.15 (-0.33; 0.02)	0.12 (-0.06; 0.29)	-0.08 (-0.26; 0.10)	-0.04 (-0.19; 0.12)
3-4	0.21 (-0.23; 0.65)	0.08 (-0.37; 0.54)	-0.02 (-0.50; 0.45)	0.04 (-0.44; 0.52)	-0.21 (-0.64; 0.23)
Continuous CAA score	-0.05 (-0.12; 0.02)	-0.07 (-0.15; 0.00)	0.04 (-0.03; 0.12)	-0.05 (-0.13; 0.03)	-0.03 (-0.10; 0.04)

Abbreviations: N, number of participants; CI, confidence interval; CAA, cerebral amyloid angiopathy.

Model 1: adjusted for age and sex.

Model 2: adjusted for age, sex, hypertension, cholesterol, lipid lowering medication, history of atrial fibrillation, antithrombotic medication and *APOE*-ε2/ε4 carriership.

[‡]Data missing for *APOE*-ε2/ε4 carriership n=35.

*P<0.05.

Supplementary Table II. The association of cerebral amyloid angiopathy score excluding cortical superficial siderosis with general cognition.

	N	MMSE Mean difference (95% CI)	N	G-factor Mean difference (95% CI)
Model 1[ǂ]	1617		1274	
CAA score excluding cSS				
0		Reference		Reference
1		-0.22 (-0.43; -0.02)*		-0.10 (-0.21; 0.00)
2-3		-0.05 (-0.36; 0.26)		-0.11 (-0.28; 0.06)
Continuous CAA score excluding cSS		-0.08 (-0.22; 0.06)		-0.07 (-0.15; 0.01)
Model 2[ǂ]	1582		1243	
CAA score excluding cSS				
0		Reference		Reference
1		-0.21 (-0.42; -0.00)*		-0.10 (-0.21; 0.01)
2-3		-0.05 (-0.36; 0.27)		-0.11 (-0.28; 0.06)
Continuous CAA score excluding cSS		-0.07 (-0.22; 0.07)		-0.07 (-0.15; 0.01)

Abbreviations: N, number of participants; MMSE, Mini Mental State Examination; CI, confidence interval; CAA, cerebral amyloid angiopathy; cSS, cortical superficial siderosis.
Model 1: adjusted for age and sex.
Model 2: adjusted for age, sex, hypertension, cholesterol, lipid lowering medication, history of atrial fibrillation, antithrombotic medication and APOE-ε2/ε4 carriership.
[ǂ]Data missing for MMSE n=5, APOE-ε2/ε4 carriership n=35, respectively for MMSE analysis. Data missing for g-factor n=348, APOE-ε2/ε4 carriership n=31, respectively for g-factor analysis.

*$P<0.05$.

Supplementary Table III. The association of cerebral amyloid angiopathy score excluding white matter hyperintensities with general cognition.

	N	MMSE Mean difference (95% CI)	N	G-factor Mean difference (95% CI)
Model 1[‡]	1617		1274	
CAA score excluding WMH				
0		Reference		Reference
1		-0.02 (-0.25; 0.21)		-0.03 (-0.15; 0.09)
2-3		0.17 (-0.58; 0.92)		0.12 (-0.28; 0.53)
Continuous CAA score excluding WMH		0.01 (-0.19; 0.21)		-0.01 (-0.12; 0.10)
Model 2[‡]	1582		1243	
CAA score excluding WMH				
0		Reference		Reference
1		-0.01 (-0.24; 0.22)		-0.03 (-0.16; 0.09)
2-3		0.15 (-0.60; 0.90)		0.10 (-0.30; 0.51)
Continuous CAA score excluding WMH		0.01 (-0.19; 0.22)		-0.01 (-0.12; 0.10)

Abbreviations: N, number of participants; MMSE, Mini Mental State Examination; CI, confidence interval; CAA, cerebral amyloid angiopathy; WMH, white matter hyperintensities.
Model 1: adjusted for age and sex.
Model 2: adjusted for age, sex, hypertension, cholesterol, lipid lowering medication, history of atrial fibrillation, antithrombotic medication and APOE-ε2/ε4 carriership.
[‡]Data missing for MMSE n=5, APOE-ε2/ε4 carriership n=35 of MMSE analysis. Missing data for g-factor n=348, APOE-ε2/ε4 carriership n=31 of g-factor analysis.
*P<0.05.

Supplementary Table IV. The association of cerebral amyloid angiopathy score excluding white matter hyperintensities with stroke, dementia and mortality.

	N	n	Stroke HR (95%CI)	n	Dementia HR (95%CI)	n	Mortality HR (95%CI)
Model 1							
CAA score excluding WMH							
0	1280	48	1.00 (reference)	59	1.00 (reference)	212	1.00 (reference)
1	318	13	1.03 (0.56–1.92)	14	0.83 (0.46–1.49)	78	1.29 (0.99–1.68)
2-3	24	1	0.80 (0.11–5.86)	4	2.22 (0.79–6.25)	8	1.13 (0.56–2.31)
Continuous CAA score excluding WMH	1622	62	0.99 (0.58–1.69)	77	1.09 (0.70–1.70)	298	1.20 (0.97–1.50)
Model 2[‡]							
CAA score excluding WMH							
0	1253	47	1.00 (reference)	56	1.00 (reference)	209	1.00 (reference)
1	310	13	1.01 (0.54–1.87)	14	0.84 (0.62–1.51)	76	1.24 (0.95–1.62)
2-3	24	1	0.66 (0.09–4.93)	4	1.77 (0.62–5.06)	8	1.02 (0.50–2.10)
Continuous CAA score excluding WMH	1587	61	0.94 (0.56–1.61)	74	1.05 (0.67–1.63)	293	1.15 (0.92–1.43)

Abbreviations: N, number of participants; n, number of events; HR, hazard ratio; CI, confidence interval; CAA, cerebral amyloid angiopathy; WMH, white matter hyperintensities.

Model 1: adjusted for age and sex.

Model 2: adjusted for age, sex, hypertension, cholesterol, lipid lowering medication, history of atrial fibrillation, antithrombotic medication and *APOE*-ε2/ε4 carriership.

[‡]Data missing for *APOE*-ε2/ε4 carriership n=35.

*P<0.05.

Supplementary Table V. The association of the cerebral amyloid angiopathy score according to the modified boston criteria with stroke, dementia and mortality.

	N	n	Stroke HR (95% CI)	n	Dementia HR (95% CI)	n	Mortality HR (95% CI)
Model 1							
CAA score according to modified Boston criteria							
0	1317	51	1.00 (reference)	59	1.00 (reference)	225	1.00 (reference)
1	202	6	0.73 (0.31–1.71)	10	0.98 (0.50–1.91)	50	1.53 (0.99–1.84)
2	103	5	1.09 (0.43–2.75)	8	1.44 (0.68–3.03)	23	1.00 (0.65–1.54)
Continuous CAA score according to modified Boston criteria	1622	62	0.96 (0.62–1.49)	77	1.15 (0.81–1.63)	298	1.08 (0.90–1.30)
Model 2‡							
CAA score according to modified Boston criteria							
0	1289	50	1.00 (reference)	56	1.00 (reference)	222	1.00 (reference)
1	197	6	0.72 (0.31–1.68)	10	0.97 (0.49–1.91)	49	1.27 (0.92–1.74)
2	101	5	0.95 (0.38–2.42)	8	1.37 (0.65–2.92)	22	0.93 (0.60–1.45)
Continuous CAA score according to modified Boston criteria	1587	61	0.91 (0.58–1.41)	74	1.13 (0.79–1.60)	293	1.04 (0.86–1.25)

Abbreviations: N, number of participants; n, number of events; HR, hazard ratio; CI, confidence interval; CAA, cerebral amyloid angiopathy.
Model 1: adjusted for age and sex.
Model 2: adjusted for age, sex, hypertension, cholesterol, lipid lowering medication, history of atrial fibrillation, antithrombotic medication and APOE-ε2/ε4 carriership.
‡Data missing for APOE-ε2/ε4 carriership n=35.
*P<0.05.

Supplementary Table VI. STROBE statement checklist(5)

	Item No	Recommendation	Page
**Title and abstract**	1	(*a*) Indicate the study's design with a commonly used term in the title or the abstract	1
		(*b*) Provide in the abstract an informative and balanced summary of what was done and what was found	2
**Introduction**			
Background/rationale	2	Explain the scientific background and rationale for the investigation being reported	3
Objectives	3	State specific objectives, including any prespecified hypotheses	3
**Methods**			
Study design	4	Present key elements of study design early in the paper	4
Setting	5	Describe the setting, locations, and relevant dates, including periods of recruitment, exposure, follow-up, and data collection	4
Participants	6	(*a*) Give the eligibility criteria, and the sources and methods of selection of participants. Describe methods of follow-up	4-7; suppl.
		(*b*) For matched studies, give matching criteria and number of exposed and unexposed	NA
Variables	7	Clearly define all outcomes, exposures, predictors, potential confounders, and effect modifiers. Give diagnostic criteria, if applicable	4-7; suppl.
Data sources/ measurement	8	For each variable of interest, give sources of data and details of methods of assessment (measurement). Describe comparability of assessment methods if there is more than one group	4-7; suppl.
Bias	9	Describe any efforts to address potential sources of bias	NI
Study size	10	Explain how the study size was arrived at	7; suppl.
Quantitative variables	11	Explain how quantitative variables were handled in the analyses. If applicable, describe which groupings were chosen and why	7-8; 11-12; suppl.
Statistical methods	12	(*a*) Describe all statistical methods, including those used to control for confounding	6-7
		(*b*) Describe any methods used to examine subgroups and interactions	6-7
		(*c*) Explain how missing data were addressed	6
		(*d*) If applicable, explain how loss to follow-up was addressed	NI
		(*e*) Describe any sensitivity analyses	7
**Results**			
Participants	13	(a) Report numbers of individuals at each stage of study—eg numbers potentially eligible, examined for eligibility, confirmed eligible, included in the study, completing follow-up, and analysed	4; suppl.
		(b) Give reasons for non-participation at each stage	4
		(c) Consider use of a flow diagram	Suppl. Figure 1

Descriptive data	14	(a) Give characteristics of study participants (eg demographic, clinical, social) and information on exposures and potential confounders	16
		(b) Indicate number of participants with missing data for each variable of interest	NI
		(c) Summarise follow-up time (eg, average and total amount)	5-6
Outcome data	15	Report numbers of outcome events or summary measures over time	5-6
Main results	16	(*a*) Give unadjusted estimates and, if applicable, confounder-adjusted estimates and their precision (eg, 95% confidence interval). Make clear which confounders were adjusted for and why they were included	8; Tables
		(*b*) Report category boundaries when continuous variables were categorized	4-5
		(*c*) If relevant, consider translating estimates of relative risk into absolute risk for a meaningful time period	8; Figure 4
Other analyses	17	Report other analyses done—eg analyses of subgroups and interactions, and sensitivity analyses	Suppl.
**Discussion**			
Key results	18	Summarise key results with reference to study objectives	10
Limitations	19	Discuss limitations of the study, taking into account sources of potential bias or imprecision. Discuss both direction and magnitude of any potential bias	11
Interpretation	20	Give a cautious overall interpretation of results considering objectives, limitations, multiplicity of analyses, results from similar studies, and other relevant evidence	10-12
Generalisability	21	Discuss the generalisability (external validity) of the study results	11
**Other information**			
Funding	22	Give the source of funding and the role of the funders for the present study and, if applicable, for the original study on which the present article is based	25

Abbreviations: suppl., supplementary appendix; NA, not applicable; NI, not included in manuscript.

Supplementary Figure

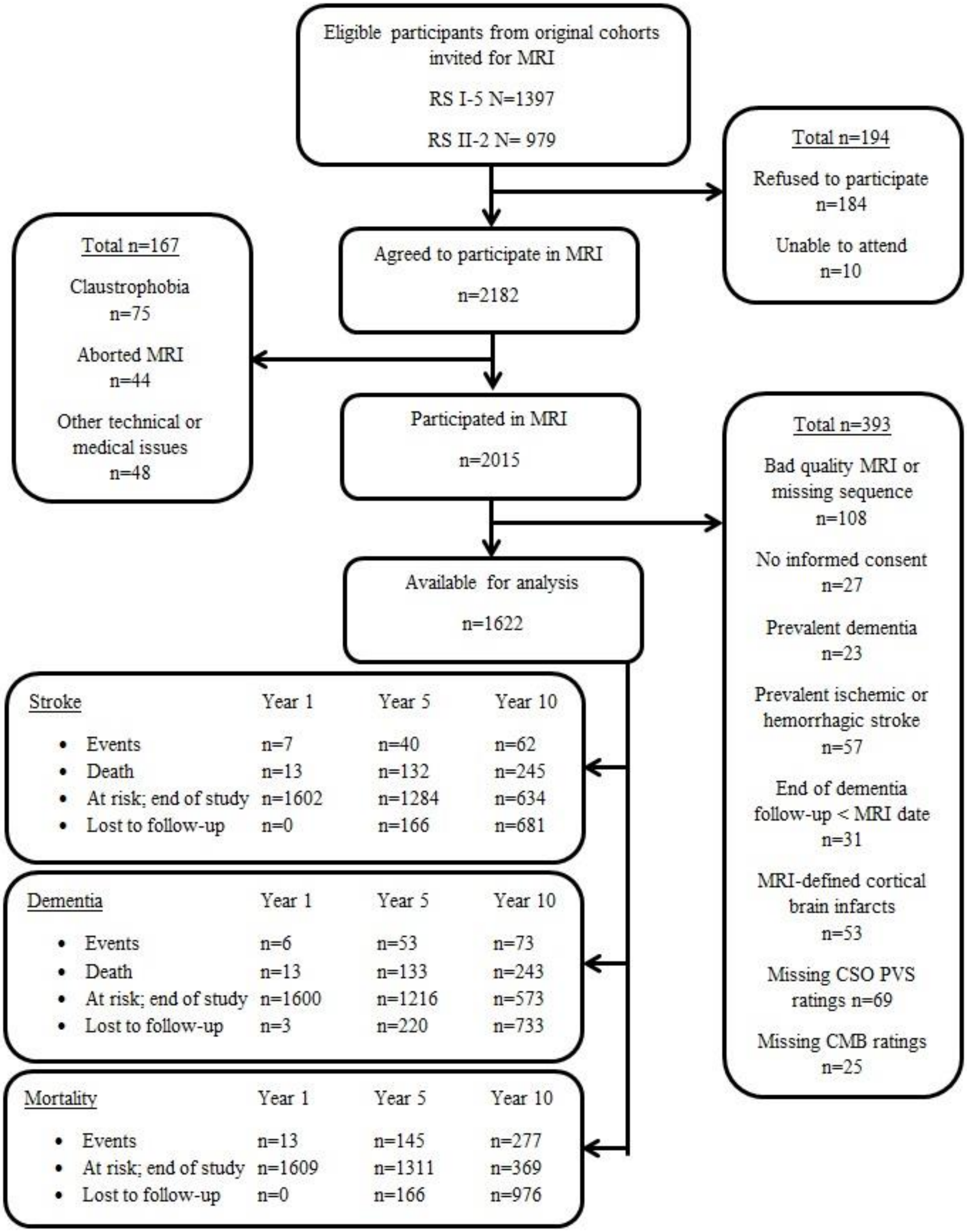

Supplementary Figure I. Flowchart of study population.

Abbreviations: MRI, magnetic resonance imaging; CSO, centrum semiovale; PVS, perivascular spaces; CMB, cerebral microbleed.
*End of follow-up for stroke and dementia was January 1st 2016, and for mortality June 16th 2017.

Supplemental References

1. Wieberdink RG, Ikram MA, Hofman A, Koudstaal PJ, Breteler MM. Trends in stroke incidence rates and stroke risk factors in Rotterdam, the Netherlands from 1990 to 2008. Eur J Epidemiol. 2012;27(4):287-95.
2. Leening MJ, Kavousi M, Heeringa J, van Rooij FJ, Verkroost-van Heemst J, Deckers JW, et al. Methods of data collection and definitions of cardiac outcomes in the Rotterdam Study. Eur J Epidemiol. 2012;27(3):173-85.
3. Wenham PR, Price WH, Blandell G. Apolipoprotein E genotyping by one-stage PCR. Lancet. 1991;337(8750):1158-9.
4. United Nations Educational SaCOU. International Standard Classification of Education (ISCED) 1976. Available from: http://unesdoc.unesco.org/images/0002/000209/020992eb.pdf.
5. von Elm E, Altman DG, Egger M, Pocock SJ, Gotzsche PC, Vandenbroucke JP. The Strengthening the Reporting of Observational Studies in Epidemiology (STROBE) statement: guidelines for reporting observational studies. Lancet. 2007;370(9596):1453-7.
